# Perovskite-Type Cu-Sn Hydroxide Microspheres as a Dual-Functional Electrocatalyst for Highly Efficient Nifedipine Sensor and Supercapacitor

**DOI:** 10.3390/ijms27073311

**Published:** 2026-04-06

**Authors:** Venkatachalam Vinothkumar, Karmegam Muthukrishnan, Al Amin, Tae Hyun Kim

**Affiliations:** 1 Korea Native Animal Resources Utilization Convergence Research Institute, Soonchunhyang University, Asan 31538, Republic of Korea; 2 Department of Chemistry, Soonchunhyang University, Asan 31538, Republic of Korea; alamindu.gets@gmail.com; 3 Centre for Smart Energy Systems, Chennai Institute of Technology, Chennai 600069, India; muthukrishnank.chem@citchennai.net

**Keywords:** perovskite architecture, CuSn(OH)_6_, coprecipitation method, nifedipine, electrochemical sensor, supercapacitor

## Abstract

An important challenge for materials researchers in the modern era is the fabrication of high-performance electrodes with novel designs and structures to enhance electrochemical sensing and energy storage performance. Recently, perovskite-structured bimetallic hydroxide materials, owing to their high conductivity, decent surface area, abundant redox activity, and good stability, have emerged as promising candidates for bifunctional electrochemical applications. In this study, we designed perovskite-type CuSn(OH)_6_ microspheres via a facile coprecipitation method for nifedipine (NFD) sensing and supercapacitors (SCs). Various characterization techniques were employed to confirm the successful synthesis of CuSn(OH)_6_. The uniform formation and distribution of CuSn(OH)_6_ within the sphere structure provide rich reactive sites and enhance structural stability, thereby improving electrochemical activity. This architecture also induces a synergistic effect between Cu and Sn, which increases conductivity and accelerates redox kinetics. Consequently, the electrode modified with CuSn(OH)_6_/GCE exhibited a wide linear concentration range of 0.4–303.3 µM and a low detection limit of 0.44 µM for NFD detection. This sensor further demonstrated superior analytical reliability, with selectivity of <5%, cycling stability of 84.79%, reproducibility of 3.3%, and recovery rates of 99.2–99.8% in the serum sample. Concurrently, the CuSn(OH)_6_/NF showcased a high specific capacitance of 514 F g^−1^ at 1 A g^−1^, good longevity of 83.05% retention after 5000 cycles, and low charge transfer resistance of 6.56 Ω and solution resistance of 1.04 Ω, validating fast ion–electron transport. These results underscore that perovskite-based CuSn(OH)_6_ is an efficient dual-functional electrocatalyst for sensitive electrochemical detection and high-performance SCs.

## 1. Introduction

Globally, the prevalence of ailments, including diabetes, heart disease, and hypertension, has increased largely due to modern lifestyles and dietary habits. Hypertension, also known as the “silent killer,” cannot be neglected given its prevalence in the current scenario because the World Health Organization recognizes hypertension as an important cause of cardiovascular mortality [1]. Given the risk factors, several drugs are administered and utilized by patients to prevent this disease in order to reduce the possibility of high fatalities. Nifedipine (NFD, 1,4-dihydro-2,6-dimethyl-4-(2-nitrophenyl)-3,5-pyridinedicarboxylic acid) is a potent calcium channel blocker that is extensively employed to treat hypertension, migraine, angina pectoris, and cardiovascular disease [1,2]. The serum drug levels vary from 10 to 100 ng mL^−1^ after the typical therapeutic dosage of NFD (30 mg) [2]. Excessive NFD usage can also cause several problems in human systems, such as dizziness, nausea, severe constipation, vomiting, flushing, pounding heartbeat, and tachycardia [3]. Hence, precise, fast, and inexpensive analytical determination of NFD quantities in biological samples is vital for health monitoring.

Numerous analytical methods, such as liquid chromatography–tandem mass spectrometry [4], spectrophotometry [5], micellar electrokinetic capillary chromatography [6], and high-performance liquid chromatography [7], have been employed to detect NFD. All of these traditional analytical approaches for NFD measurement often need complex sample preparation procedures before analysis, rendering the entire process time-consuming and unsuitable for regular sample analysis [1]. In contrast, electrochemical methods are well known for their excellent selectivity, high sensitivity, low cost, quick analysis time, wide dynamic range, and rapid reaction response [8]. These advantageous properties make electrochemical techniques a better alternative to previously established conventional approaches for NFD analysis [1,3]. In this context, various electrochemical platforms based on different electrode materials have been developed for NFD sensing, which include Ag-nanoparticle-modified glassy carbon electrode (0.72 µM) [9], boron-doped diamond electrode (0.612 µM) [10], carboxylic acid-functionalized multiwalled carbon nanotubes modified with polyaniline nanoparticles assembled on ITO plates (1.0 µM) [11], and mercury meniscus silver solid amalgam electrode (1.2 µM) [12]. However, all these NFD sensors had high detection limits and negligible interference in complex matrices. Therefore, it is important and remains challenging in practical use to propose innovative, highly efficient, and cost-effective nanomaterials for NFD detection.

Simultaneously, the pressing demand for alternative, sustainable energy storage technologies has been prompted by rising worldwide energy consumption, fast advances in technology, and the rapid depletion of fossil fuel resources [13]. Supercapacitors (SCs) are a form of energy storage system that have drawn significant attention because of their environmental friendliness, safe operation, low cost, fast charge–discharge capability, long cyclic life, and high power density [13,14]. These features have expanded their use in smart vehicles, portable electronics, and wearable devices. Nevertheless, their low energy density and high self-discharge pose a major obstacle to wider adoption [15]. To overcome these difficulties and increase the scalability of SCs, new electrode materials with improved capacitive characteristics are urgently required to meet energy density demands.

Pseudocapacitive-type SCs are a better choice to address these limitations, as electrochemical energy is stored through fast faradaic redox reactions at the electrode–electrolyte interface, achieving higher energy density than electric double-layer capacitive (EDLC)-type SCs [15,16,17]. In the recent past, transition metal hydroxides/oxides have been proven to be promising electrode materials for electrochemical sensors and SCs due to their natural abundance, cost-effectiveness, environmentally benign nature, and good electrochemical properties [8,18,19]. Metal hydroxide stannates (MSn(OH)_6_, M = Zn, Cu, and Co) are members of the perovskite family and have garnered a lot of interest as sensors and SC electrodes, thanks to their simple synthesis routes, tunable characteristics, high theoretical capacitance, low production costs, and high reproducibility [8,20,21]. Because of its adjustable structure, high redox activity, variable morphology, low cost, ease of synthesis, reduced bulk, and improved surface area, copper hydroxide stannate (CHS, CuSn(OH)_6_), which belongs to the perovskite group with a ReO_3_ structure [22], has recently been demonstrated as an excellent electrode material for sensors [23], photocatalysts [24], lithium ion batteries [25], and SCs [21]. The unique nanoarchitecture of the CHS material, identified by easily accessible active sites, multiple valence states, and numerous pores for rapid ion transfer, can significantly improve efficiency [23]. The strong chemical stability and structural flexibility of CHS also enhance its performance in sensing and SCs. Considering the use of CHS, J. Li et al. developed a CHS–MWCNT–βCD/p-Arg hybrid electrode for indole-3-lactic acid [23]. A CHS/CNF-modified electrode was employed for NO_2_ gas sensors and SCs by A. V. Loginov et al. [21]. Nonetheless, these electrodes exhibited limited sensitivity, poor selectivity, low specific capacitance, and inferior stability. As a consequence, electrochemical applications based on CHS electrodes have not been extensively studied. Furthermore, from a fabrication standpoint, using a coprecipitation approach has several benefits, such as shorter synthesis times, greater energy efficiency, and the possibility of bulk production, although it has limited control over particle shape and size [17,20]. Despite complicated multi-step techniques, this approach is straightforward, inexpensive, and versatile, addressing the practical necessities for sensor and SC electrode fabrication. The efficient introduction of Cu and Sn hydroxide into this perovskite matrix thus promises a novel and affordable strategy for constructing high-performance sensor and SC electrode materials. Accordingly, the synthesis, characterization, and optimization of CHS materials have emerged as a significant research priority.

In light of the above discussion, this study presents a cost-effective and environmentally friendly synthesis approach using a facile coprecipitation route to fabricate CuSn(OH)_6_ microspheres for NFD sensing and SCs. The structural and morphological properties of the synthesized material were characterized using XRD, BET, FESEM, EDX, and TEM analyses, which confirmed the successful formation of CuSn(OH)_6_. Subsequently, the electrochemical behavior of the perovskite electrode was evaluated for dual applications: supercapacitor performance and NFD sensing. The inherent structural and electrochemical properties of the perovskite-type architecture account for its unique dual functionality. The CuSn(OH)_6_ microspheres possess a uniform distribution and abundant -OH active sites, allowing for faster ion diffusion and electron transfer, which improves NFD sensing and SC capability. The optimized modified sensor displayed a low detection limit, good selectivity, high stability, and dependable recovery range in a serum sample, highlighting its superior analytical performance toward NFD. On the other hand, the CuSn(OH)_6_ electrode exhibited efficient charge transfer and enhanced redox activity, resulting in high capacitance, better durability, and low resistance, demonstrating potential for high-performance SCs. Thus, the integration of perovskite bimetal hydroxide provides a sustainable synthetic pathway for making efficient electrode materials for both energy storage and sensing applications.

## 2. Results and Discussion

### 2.1. Material Characterization

#### 2.1.1. Textural Analysis of the Perovskite

**Figure 1:**
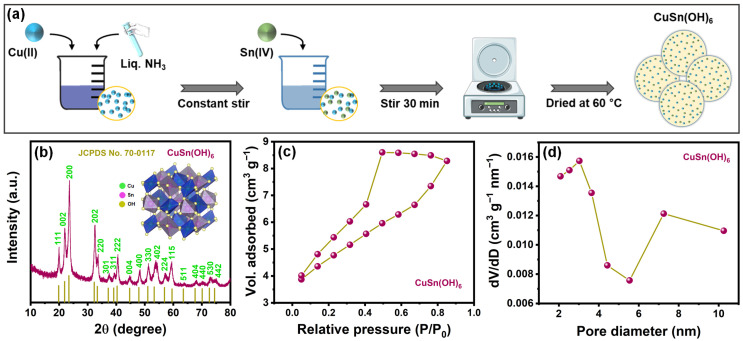
(**a**) Schematic diagram of CuSn(OH)_6_ synthesis, (**b**) XRD pattern (crystallographic structure of perovskite-type CuSn(OH)_6_), (**c**) nitrogen sorption isotherm, and (**d**) pore size distribution curve of CuSn(OH)_6_.

Figure 1a provides a schematic illustration of the synthesis of perovskite-type CuSn(OH)_6_. Cu(II) and Sn(IV) precursors were reacted in Liq. NH_3_ solution to form the CuSn(OH)_6_ structure. The crystallinity, phase purity, and composition of the perovskite material were examined utilizing powder XRD. The diffraction signals (Figure 1b) observed at 19.83°, 21.92°, 23.43°, 32.3°, 33.38°, 37.21°, 39.13°, 40.29°, 44.69°, 47.92°, 51.07°, 53.29°, 56.91°, 59.54°, 63.52°, 67.61°, 70.11°, 72.64°, and 74.46° were identified as the crystal planes (111), (002), (200), (202), (220), (301), (311), (222), (004), (400), (330), (402), (224), (115), (511), (404), (440), (530), and (442), respectively. These signals are evidence of the CuSn(OH)_6_ phase (JCPDS No. 70-0117) [22], which demonstrates that the perovskite structure was successfully fabricated. The sharp and well-resolved peaks without an impurity phase further confirm the high purity, crystallinity, and composition of CuSn(OH)_6_. BET analysis was employed to assess key structural characteristics (specific surface area and pore size distribution) in the developed CuSn(OH)_6_ sample. The nitrogen adsorption–desorption isotherm (Figure 1c) for CuSn(OH)_6_ showed a type IV profile with a visible hysteresis loop at a relative pressure of 0.4–0.9, signifying a mesoporous structure. The BET specific surface area was 15.34 m^2^ g^−1^. Combining with the BJH plot (Figure 1d), the pore size distribution implies about 3 nm. These textural properties suggest that the perovskite CuSn(OH)_6_ is anticipated to improve electrochemical activity by offering plentiful redox active sites (Cu/Sn metal centers and -OH groups), promoting ion transport, and facilitating efficient charge transfer, which makes it a potential electrode material for electrochemical applications.

#### 2.1.2. Morphological Analysis of CuSn(OH)_6_

**Figure 2:**
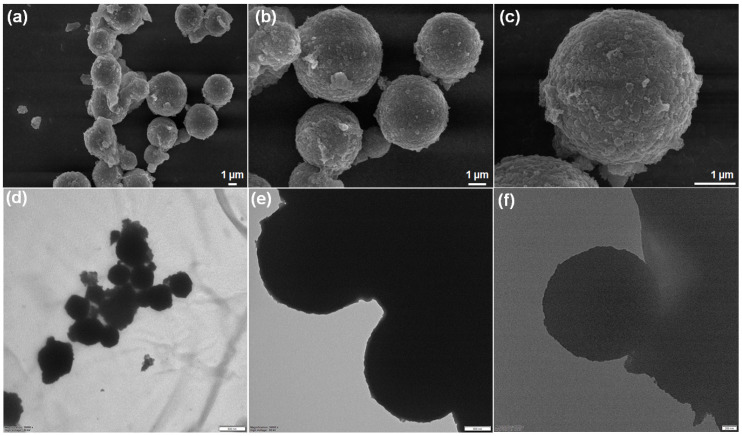
(**a**–**c**) FESEM and (**d**–**f**) TEM images of CuSn(OH)_6_ at different magnifications.

**Figure 3:**
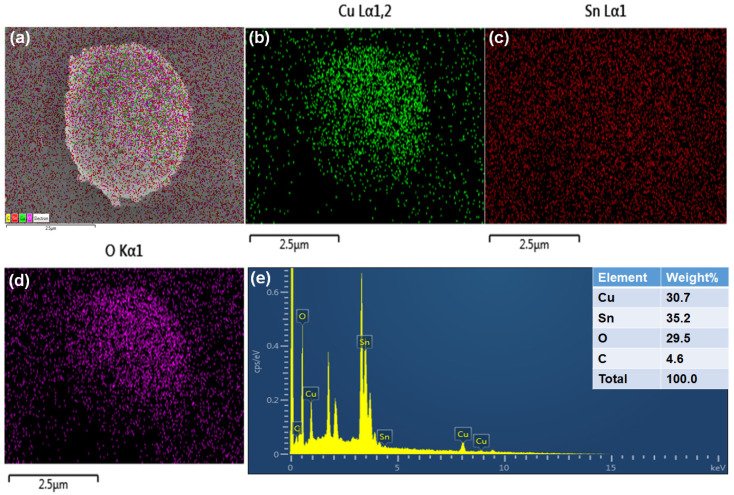
(**a**–**d**) Elemental mapping images and (**e**) EDX spectrum of CuSn(OH)_6_.

Figure 2 presents the FESEM and TEM morphological characterization of the synthesized sample. Figure 2a–c shows the FESEM images of CuSn(OH)_6_, unveiling a sphere-like structure with the arrangement of tiny particles at high to low magnifications. TEM images also confirm that the sample architecture has a sphere (Figure 2d,e), which was successfully developed through a facile coprecipitation method. The unique structural formation not only provides abundant reactive sites and high conductivity but also more reaction paths for ion and electron transport, enhancing the electrochemical performance of the electrode material. Moreover, to verify the uniform distribution within the structure, elemental mapping and the EDX spectrum for CuSn(OH)_6_ were analyzed. The elemental mapping proved that Cu, Sn, and O elements were homogeneously distributed throughout the CuSn(OH)_6_ sphere (Figure 3a–d). The EDX spectrum in Figure 3e confirms the existence of material constituents, representing that the sphere architecture grew successfully. In addition, the C element was detected in the sample due to the adsorption of atmospheric carbon. These morphological studies support that the sphere CuSn(OH)_6_ system is more beneficial for electrochemical reactions.

### 2.2. Electrochemical Sensing of CuSn(OH)_6_/GCE

#### 2.2.1. Voltammetric Behavior of NFD

**Figure 4:**
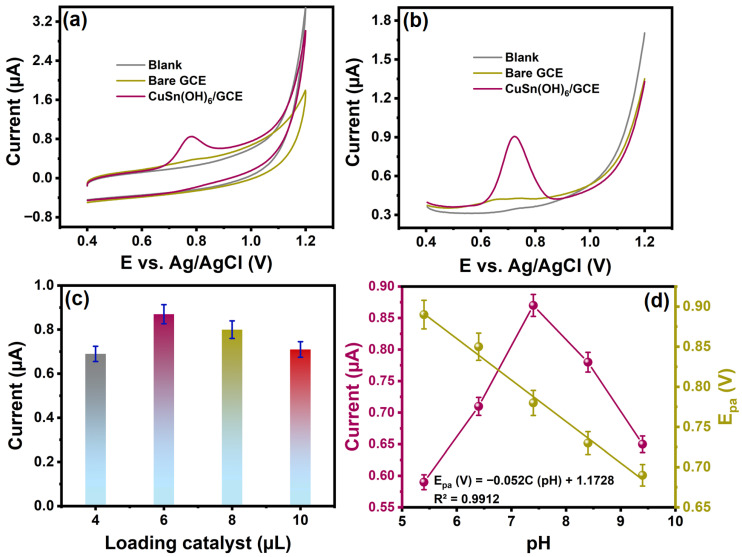
(**a**) CV and (**b**) DPV oxidation curves of blank, bare, and modified CuSn(OH)_6_ electrodes in 0.1 M PBS at pH 7.4 towards 20 µM NFD, (**c**) CV oxidation profiles of NFD on the modified sensor with various loading amounts, and (**d**) the dependence of the reaction on the pH was recorded at different pH values in the presence of 20 μM NFD using CuSn(OH)_6_/GCE.

The CV behavior of NFD oxidation at bare and modified CuSn(OH)_6_/GCE was studied at pH 7.4 and a scan rate of 50 mV s^−1^, as presented in Figure 4a. The blank modified electrode showed no CV response, suggesting the absence of NFD. When 20 µM of NFD was introduced to the PBS, the bare GCE exhibited a weak oxidation current (0.41 µA) at 0.8 V due to the sluggish electron transport to the NFD. By contrast, the modified CuSn(OH)_6_/GCE demonstrated a higher oxidation current (0.87 µA) with reduced peak potential at 0.78 V, which implies that the successful modification and uniform composition of the perovskite hydroxide efficiently accelerated electron transfer processes toward NFD. The reverse scan further displayed no reduction peak for NFD in the same potential range (0.4–1.2 V), confirming that the electrochemical NFD detection is irreversible [1]. Additionally, DPV behavior (Figure 4b) of the as-proposed material and the bare electrode was examined for NFD for the first time, considering that it is a highly sensitive and effective method with low background current. An inconspicuous peak was seen at bare GCE (0.43 µA) at about 0.74 V. Remarkably, CuSn(OH)_6_/GCE delivers a significantly higher DPV signal (0.91 µA) at 0.72 V than that of the bare electrode, resulting in excellent sensing performance for NFD oxidation. The improvement in oxidation current and potential at the CuSn(OH)_6_/GCE surface is attributed to available reactive sites, good surface features, multi-valence states, and synergistic effects, all of which facilitate charge transfer for NFD electrooxidation.

#### 2.2.2. The Optimum Detection Conditions

The voltammetric signal of NFD could be influenced by the loading amount of the CuSn(OH)_6_ catalyst. Figure 4c depicts the CVs of 20 µM NFD on CuSn(OH)_6_/GCE with different drop-cast amounts (4, 6, 8, and 10 μL). The oxidation current of CuSn(OH)_6_/GCE increased up to 6 µL, representing improved interfacial kinetics at the electrode and NFD. When increasing the modification amount above 6 µL, the oxidation current decreased. This is because an excessive amount of thick film coating limits electron transfer and decreases NFD activity. Hence, 6 µL was chosen as the optimal modified dosage of CuSn(OH)_6_ for the NFD electrochemical investigation. On the other hand, the electrooxidation behavior of NFD was considerably affected by the pH of the electrolyte solutions. We assessed the effect of PBS at various pH ranges (5.4–9.4) on the oxidation current of 20 µM NFD, as shown in Figure 4d. As the pH value rose from 5.4 to 7.4, the oxidation current of NFD at CuSn(OH)_6_/GCE increased and attained its highest value at pH 7.4, which suggests proficient proton-coupled electron transfer for NFD detection [1,26]. The oxidation current rapidly dropped when the pH increased from 8.4 to 9.4 due to limited proton availability, thereby diminishing NFD performance in alkaline medium. Moreover, as pH increased, the peak potential moved to positive values, indicative of proton transfer participating in the electrochemical oxidation of the NFD [26]. The linear plot slope of the oxidation peak potential versus pH was found as −0.052 V pH^−1^ for NFD (Figure 4d). This slope is close to the Nernstian theoretical value of −0.059 V pH^−1^, proving that the number of protons and electrons transferred was the same. The m/n of NFD was further calculated according to Equation (1) [8]:

(1) $$\frac{{dE}_{p}}{d_{p}H}=\frac{-2.303mRT}{nF}$$

where m and n denote the number of protons and electrons involved, and other values are constants. The value of m/n in the NFD detection was estimated to be 0.88, which is approximately equivalent to 1, confirming that the protons (H^+^) and electrons (e^−^) equally contributed to the NFD oxidation [3,8,20]. This result agrees with previous works, reporting that the CuSn(OH)_6_ sensor involves 2H^+^ and 2e^−^ transfer toward NFD oxidation [1,3,26]. Therefore, a pH of 7.4 was discovered to be ideal for NFD, and it was employed for the subsequent voltammetric study.

#### 2.2.3. Scan Rate Effect and Reaction Kinetics

**Figure 5:**
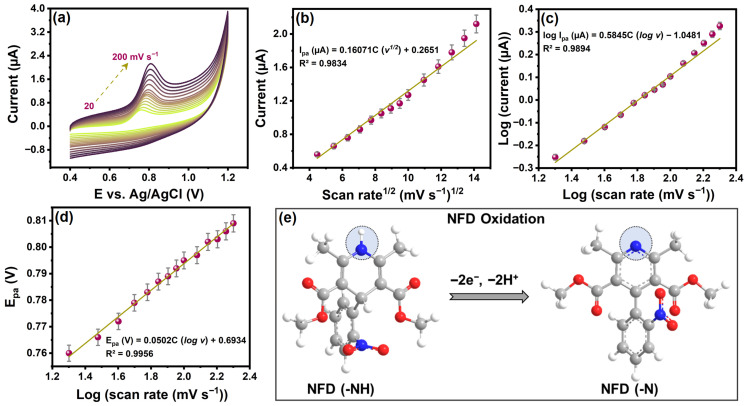
(**a**) CVs of CuSn(OH)_6_/GCE at different scan rates for 20 µM NFD, (**b**) the linear relationship between peak current and square root of the scan rates, (**c**) the calibration plots of log peak current versus log scan rates, (**d**) peak potential versus log scan rates, and (**e**) the plausible electrochemical oxidation mechanism of NFD.

To elucidate the rate-determining step and reaction kinetics in the electrochemical irreversible oxidation of NFD, CV curves of CuSn(OH)_6_/GCE were recorded at pH 7.4 at different scan rates from 20 to 200 mV s^−1^. As seen in Figure 5a, the oxidation peak of NFD rose proportionally with the increase in the scan rate. The linear plot (Figure 5b) of the oxidation current versus the square root of the scan rate yields a good regression equation: I_pa_ (µA) = 0.16071C (v^1/2^) + 0.2651 (R^2^ = 0.9834). This revealed that the NFD oxidation of CuSn(OH)_6_/GCE was diffusion-controlled kinetics [9]. The calibration curve between log oxidation current and log scan rate (Figure 5c) further verified this statement. The linear regression equation for log I_pa_ (µA) was 0.5845C (log v)–1.0481 (R^2^ = 0.9894). The detected slope of 0.58 is equivalent to the theoretical value of 0.5 [27], which supports a diffusion process. In addition, the oxidation peak potential shifted slightly to positive directions when the scan rate was raised, probably due to the polarization effect attributed to the irreversibility of the oxidation process. The linear relation of oxidation peak potential versus log scan rate is depicted in Figure 5d, with the regression equation E_pa_ (V) = 0.0502C (log v) + 0.6934 (R^2^ = 0.9956). For the irreversible oxidation process of NFD, the Laviron equation (Equation (2)) [27] was utilized to measure the number of electrons (n) transferred.

(2) $$E_{pa}\left(0.0502\right)=\frac{2.303RT}{\alpha nF}$$

In this equation, α is considered as 0.5 for irreversible, while the remaining values are constants. The n transfer during NFD oxidation was measured to be 2.35 (close to 2). This conclusion authenticates that the electrochemical oxidation processes of NFD on CuSn(OH)_6_/GCE entailed 2H^+^ and 2e^−^ [9], and the NFD reaction mechanism is proposed in Figure 5e.

#### 2.2.4. DPV Method Analysis of NFD at CuSn(OH)_6_/GCE

**Figure 6:**
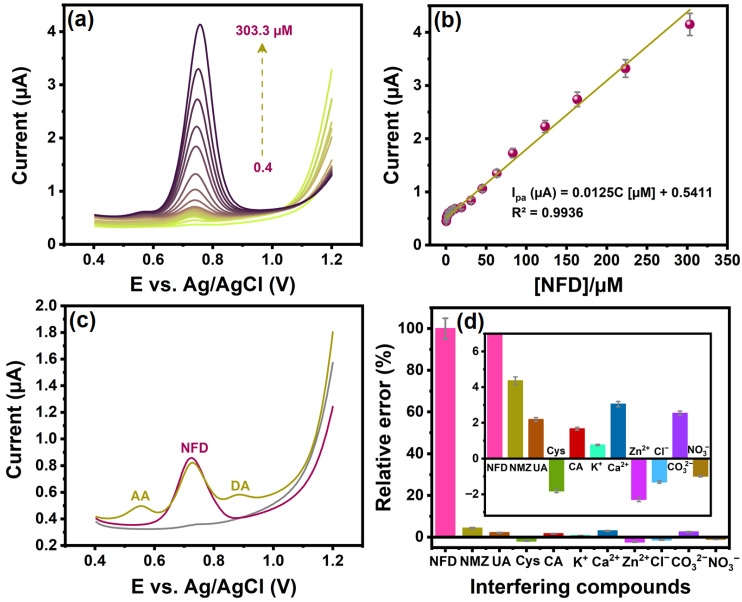
(**a**) DPV responses of the CuSn(OH)_6_/GCE to NFD at increasing concentration ranges from 0.4 to 303.3 µM, (**b**) the calibration curve relates current to NFD concentrations, (**c**) DPV curves of the modified sensor recorded for NFD in the presence of AA and DA, and (**d**) DPV studies of CuSn(OH)_6_/GCE to detect NFD in the presence of other potentially interfering species such as NMZ, UA, Cys, CA, K^+^, Ca^2+^, Zn^2+^, Cl^−^, CO_3_^2−^, and NO_3_^−^.

The DPV method was applied to assess the analytical performance of the CuSn(OH)_6_ sensor for NFD oxidation owing to its low detection limit, wide linearity, and high selectivity. Figure 6a reveals the DPV profiles recorded at pH 7.4 with varying NFD concentrations using CuSn(OH)_6_/GCE. The oxidation current was directly proportional to NFD concentrations in the range from 0.4 to 303.3 µM using the optimized conditions. The regression equation of the linear calibration curve (Figure 6b) is I_pa_ (µA) = 0.0125C [µM] + 0.5411 (R^2^ = 0.9936). The limit of detection (LOD) of NFD was calculated using Equation (3) [8]:

(3) $$LOD=3\times \frac{Standard\;deviation}{Slope\;of\;the\;linear\;curve}$$

Based on this calibration result, the LOD of NFD on CuSn(OH)_6_/GCE was 0.44 µM, and the estimated sensitivity was 0.173 µA µM^−1^ cm^−2^ (using slope/electrode surface area) [8]. Table 1 provides a detailed comparison of the results of this sensor with those of previously reported sensors for NFD. When compared to various previously documented electrodes, the CuSn(OH)_6_/GCE exhibited a low LOD and a wide linear range compared to NFD. Notably, the one-pot synthesis of the perovskite-type CuSn(OH)_6_ assembly is a more facile, straightforward, and cost-effective approach than the reported modified materials, making it favorable for electrochemical applications. Furthermore, the homogeneous arrangement of CuSn(OH)_6_ microspheres offers catalytically active sites and channels, as well as better conductivity, which enhances electron transport towards NFD oxidation.

#### 2.2.5. Study of Selectivity, Stability, and Reproducibility

Studies on selectivity, stability, and reproducibility have significance for developing reliable electrochemical sensors. The influence of several potential interferents was investigated by the DPV technique to evaluate the selectivity of CuSn(OH)_6_/GCE for 20 µM NFD detection at pH 7.4. The oxidation response of NFD with simultaneous interfering molecules, including ascorbic acid (AA) and dopamine (DA), was studied in Figure 6c. The peak currents of AA and DA did not alter the NFD oxidation behavior, evidence that the designed perovskite sensor is selective and suitable for analyzing these compounds simultaneously. Also, the CuSn(OH)_6_/GCE sensor selectivity study was carried out in the presence of possibly interfering substances for 20 µM NFD (Figure 6d). A 10-fold concentration of bioactive molecules such as nimesulide (NMZ), uric acid (UA), L-cysteine (Cys), and caffeic acid (CA) and a 20-fold concentration of common metal species such as K^+^, Ca^2+^, Zn^2+^, Cl^−^, CO_3_^2−^, and NO_3_^−^ were tested and all showed no notable change in NFD peak current, which confirms the good anti-interference ability of CuSn(OH)_6_/GCE. The relative error of less than 5% further demonstrates the excellent selectivity of the modified CuSn(OH)_6_ sensor (Figure 6d inset).

**Figure 7:**
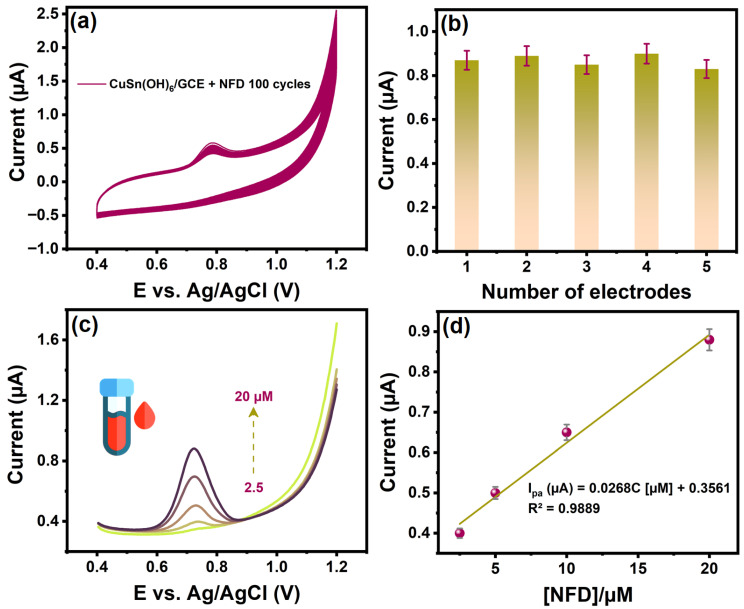
(**a**) Scan rate of 50 mV s^−1^ at CuSn(OH)_6_/GCE with addition of NFD for 100 cycles, (**b**) CV response using the five different prepared electrodes for NFD, (**c**) DPV curves of CuSn(OH)_6_/GCE in the serum sample with various concentrations of NFD, and (**d**) linear plot of oxidation current versus NFD spiked additions.

The cyclic stability of CuSn(OH)_6_/GCE was performed over 100 cycles to determine the long-term efficiency for NFD. After 100 successive cycles (Figure 7a), the NFD oxidation remained at 84.79% of its initial response, reflecting considerable durability. Subsequently, the reproducibility of the proposed sensor was tested by five independently prepared electrodes to detect NFD under the same optimal parameters. The relative standard deviation (RSD) of these five replicate measurements for NFD was 3.3% (Figure 7b), highlighting high reproducibility. These findings showcased that the developed sensor possessed the desired selectivity, stability, and reproducibility.

#### 2.2.6. Real Sample Analysis

The well-established DPV system was implemented to evaluate the feasibility and viability of CuSn(OH)_6_/GCE for detecting NFD in the serum sample. Serum was purchased from Sigma-Aldrich, and an appropriate quantity was diluted with 0.1 M PBS (pH 7.4). Known NFD concentrations of 2.5, 5, 10, and 20 µM were added to the electrochemical cell. With each addition of NFD, a well-defined oxidation peak was detected, and it was proportional to the concentration, resulting in a good linear plot with R^2^ = 0.9889 (Figure 7c,d). Recovery rates for the serum sample were 99.2–99.8%, and RSD values ranged from 2.08 to 2.31%. These results proved that the well-known method with the CuSn(OH)_6_ sensor is reliable and accurate for the assay of NFD in biological matrix samples.

### 2.3. Electrochemical Supercapacitive Performance of CuSn(OH)_6_/NF

#### 2.3.1. Cyclic Voltammetry Analysis

**Figure 8:**
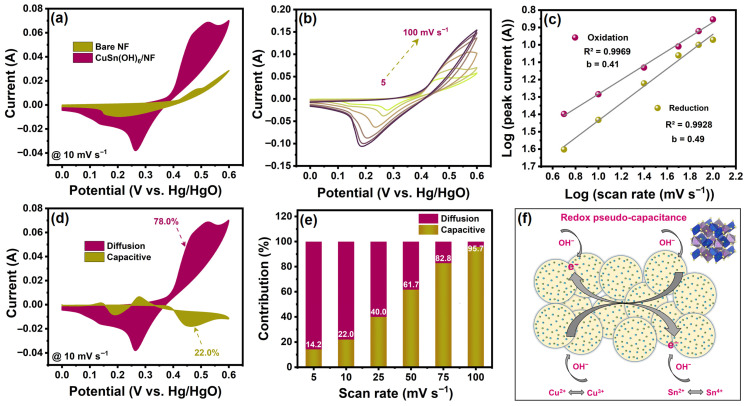
(**a**) Comparative CV graph of bare NF and fabricated CuSn(OH)_6_/NF in 3 M KOH at a scan rate of 10 mV s^−1^, (**b**) CV profiles of CuSn(OH)_6_/NF at scan rates from 5 to 100 mV s^−1^, (**c**) plot of log peak current versus log scan rates, (**d**) the capacitive and diffusion contributions for CuSn(OH)_6_/NF at 10 mV s^−1^, (**e**) the capacitive and diffusion contribution ratios at various scan rates, and (**f**) CuSn(OH)_6_/NF electrode working redox mechanism.

The CV technique was employed to examine the electrochemical supercapacitive performance of the bare NF and fabricated CuSn(OH)_6_/NF in 3 M KOH using a three-electrode cell. The CV curves of CuSn(OH)_6_/NF at 10 mV s^−1^ (Figure 8a) revealed a larger geometric area and a higher peak current than the bare NF. This is due to the high electrical conductivity, improved surface area, and excellent redox activity of the perovskite hydroxide material, which results in a higher specific capacitance. Figure 8b showcases the CV curves of CuSn(OH)_6_/NF recorded in the potential range of 0–0.6 V at diverse scan rates of 5–100 mV s^−1^. The redox peak current of the CV curves increased linearly as scan rates increased, implying reversible faradaic reactions (Cu^2+^/Cu^3+^ and Sn^2+^/Sn^4+^) attributed to the pseudocapacitive features of the electroactive material [17,28]. Equations (4)–(11) represent the associated faradaic redox processes:

(4) $${CuSn(OH)}_{6}+4{OH}^{-}↔{Cu(OH)}_{4^{2-}}+{Sn(OH)}_{6^{2-}}$$

(5) $${Cu(OH)}_{4^{2-}}↔{Cu(OH)}_{2}+{2OH}^{-}$$

(6) $${Sn(OH)}_{6^{2-}}↔{Sn(OH)}_{4}+{2OH}^{-}+{2e}^{-}$$

(7) $${Sn(OH)}_{4}↔{Sn(OH)}_{2}+{2OH}^{-}+{2e}^{-}$$

(8) $${Cu(OH)}_{2}+{OH}^{-}↔CuOOH+H_{2}O+e^{-}$$

(9) $$CuOOH+{OH}^{-}↔{CuO}_{2}+H_{2}O+e^{-}$$

(10) $${Sn(OH)}_{2}+{OH}^{-}↔SnOOH+H_{2}O+e^{-}$$

(11) $$SnOOH+{OH}^{-}↔{SnO}_{2}+H_{2}O+e^{-}$$

To further understand the mechanism of charge storage and kinetics of the CuSn(OH)_6_/NF electrode, CV was studied with various scan rates based on the power-law linear relation in Equations (12) and (13) [15]:

(12) $$I={a\times v}^{b}$$

(13) log I=loga+b log v 

here, I, v, a, and b represent the current, scan rate, and constant parameters. The charge storage kinetics generally involve capacitive-controlled or diffusion-controlled behavior. The capacitive-controlled behavior is identified by b = 1, while the diffusion-controlled behavior is identified by b = 0.5 [19]. The CuSn(OH)_6_/NF yielded b values of 0.41 (oxidation) and 0.49 (reduction), as determined by the slope of the linear plot of log v versus log i (Figure 8c). The identified values equal 0.5, so the charge storage kinetics of CuSn(OH)_6_/NF is a diffusion-controlled behavior [14,16], indicating that the electrode material is pseudocapacitive. The capacitive-controlled and diffusion-controlled contribution ratios of the CuSn(OH)_6_/NF electrode were further determined using the Trasatti and Dunn equations below (Equations (14) and (15)) [15]:

(14) $$I\left(V\right)=k_{1}v+k_{2}v^{1/2}$$

(15) $$I\left(V\right)/v^{1/2}=k_{1}v^{1/2}+k_{2}$$

where k_1_ and k_2_ are constants, whereas k_1_v and k_2_v^1/2^ are related to the capacitive and diffusion ratios. At the scan rate of 10 mV s^−1^ (Figure 8d), the capacitive and diffusion contribution ratios were 22.0% and 78.0% of the total capacitance for CuSn(OH)_6_/NF, due to the strong influence of the diffusion charge storage mechanism at lower scan rates. The capacitive contribution ratio was the highest (95.7%), and the diffusion contribution ratio was the lowest (4.3%) as the scan rate rose to 100 mV s^−1^. Consequently, the contribution ratio of the capacitive behavior gradually increased, while diffusion behavior reduced as the scan rate reached high levels (Figure 8e), because the electrolyte ions were limited at the CuSn(OH)_6_/NF electrode surface, diminishing the contribution percentages of the diffusion-governed (faradaic) processes [15]. Integration of Cu into Sn hydroxide results in a very dense sphere with a roughened surface, which broadens the number of surface active sites and offers a shortcut for the ion diffusion pathway (Figure 8f).

#### 2.3.2. Galvanostatic Charge–Discharge Analysis

**Figure 9:**
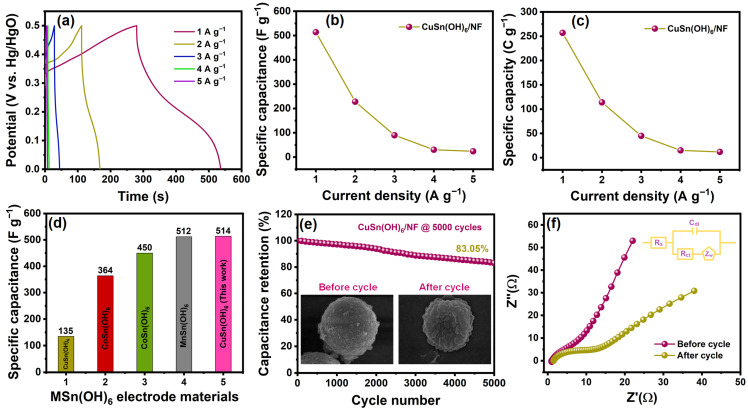
(**a**) GCD curves of CuSn(OH)_6_/NF at different charge–discharge currents of 1–5 A g^−1^, (**b**) C_sp_, and (**c**) C_s_ versus current density plots, (**d**) comparison of C_sp_ values of CuSn(OH)_6_/NF with previous reports, (**e**) long-term cyclic stability of the material over 5000 cycles (inset showing before and after cycling of FESEM images), and (**f**) Nyquist plots of CuSn(OH)_6_/NF before and after cyclic tests (inset displaying equivalent circuit diagram).

Figure 9a depicts the GCD profiles of the CuSn(OH)_6_/NF electrode measured in the potential range of 0–0.5 V at different current densities of 1–5 A g^−1^. The non-linear shapes of all GCD curves demonstrate good faradaic efficiency and superior reversibility of the pseudo-type electrode material [16,19]. The longer charging–discharging times imply higher capacitance at 1 A g^−1^, which decreases as current density increases due to the restricted diffusion of ions. The specific capacitance (C_sp_) and specific capacity (C_s_) for CuSn(OH)_6_/NF were estimated by using Equations (16) and (17) [14]:

(16) $$C_{sp}=\frac{I\times \Delta t}{m\times \Delta V}$$

(17) $$C_{s}=\frac{I\times \Delta t}{m}$$

where I (A), Δt (s), m (mg), and ΔV (V) are the current, discharge time, mass of active material, and potential window. The C_sp_ values calculated from GCD measurements for CuSn(OH)_6_/NF at 1, 2, 3, 4, and 5 A g^−1^ were 514, 228, 90, 30.4, and 24 F g^−1^ (Figure 9b). Similarly, the C_s_ values were 257, 114, 45, 15.2, and 12 F g^−1^ (Figure 9c), respectively. At high current densities, the CuSn(OH)_6_/NF electrode is limited by ion diffusion constraints within the microsphere framework, as electrolyte ions cannot effectively access internal active sites due to shorter diffusion time, resulting in slower charge storage kinetics and decreased capacitance. Conversely, CuSn(OH)_6_/NF exhibits fast ion–electron transport at low current densities owing to efficient faradaic reactions, which allows for the full exploitation of active sites. As a result, the CuSn(OH)_6_/NF electrode achieved a notable C_sp_ (514 F g^−1^), which surpassed that of other comparable metal tin hydroxide and metal tin oxide electrodes (Figure 9d and Table 2). The enhancement in C_sp_ is due to its high conductivity, accessible active sites, and uniform morphology with porous nature, which allows OH^−^ ions to easily penetrate the electrode material (CuSn(OH)_6_/NF) during faradaic reactions. The improved surface area and synergistic interactions within the perovskite structure further increase electrical conductivity and facilitate charge transport.

#### 2.3.3. Cycling Stability and Impedance Analysis

Cycling efficiency is the most important for determining the viability of SCs. To verify the cycling proficiency of the electrode, the stability test for CuSn(OH)_6_/NF was carried out via the GCD over 5000 cycles at a current density of 5 A g^−1^. After 5000 unremitting cycles as seen in Figure 9e, the modified electrode preserved 83.05% of its initial capacitance, showing a satisfactory longevity of CuSn(OH)_6_/NF. Successively, after a period of cycling, the coulombic efficiency (η) of CuSn(OH)_6_/NF remained at 97.81% under high-current-density conditions, such as 5 A g^−1^, which proves the superior long-term efficacy of the proposed electrode. The before- and after-cycling FESEM images for CuSn(OH)_6_ are shown in the Figure 9e inset. The sphere shape was slightly aggregated and collapsed during the cycling due to the successive redox reactions. This change reduces overall specific surface area whilst partly restricting ion diffusion paths, which leads to increased resistivity and slowed electrochemical kinetics. But the overall morphology remained sphere-like, demonstrating that the CuSn(OH)_6_ sphere structure remains intact even after a long cycle.

The impedance characteristics of the as-fabricated CuSn(OH)_6_/NF electrode were explored by executing EIS at frequencies from 0.01 Hz to 100 kHz and an amplitude of 5 mV. The charge transfer resistance (R_ct_), solution resistance (R_s_), Warburg resistance (Z_W_), and double-layer capacitance (C_dI_) parameters were fitted using the equivalent circuit, which is illustrated in the Figure 9f inset. The Nyquist plot of CuSn(OH)_6_/NF displays that the diameter of the semicircle in the high-frequency region correlates to R_ct_, and the *x*-axis relates to R_s_. Figure 9f exhibits the before- and after-cycling EIS curves for CuSn(OH)_6_/NF. In this analysis, the R_ct_ values were 6.56 Ω and 10.26 Ω, whilst the R_s_ values were 1.04 Ω and 1.50 Ω. Comparative EIS results of before and after the cycling test implied considerable changes in both R_ct_ and R_s_. Due to the consecutive cycling during the redox process, the electrode kinetics deteriorated, resulting in increased resistance. However, lower values of R_ct_ and R_s_ confirm the excellent durability of CuSn(OH)_6_/NF. These results highlight the potential of the perovskite-structured CuSn(OH)_6_ microspheres as a high-performance candidate for next-generation energy storage devices. By leveraging these characteristics, CuSn(OH)_6_ microspheres work as a bifunctional platform, delivering the benefit of combining sensing and supercapacitor functions into one system.

## 3. Materials and Methods

### 3.1. Materials

Copper sulfide pentahydrate (CuSO_4_.5H_2_O), sodium stannate trihydrate (Na_2_SnO_3_.3H_2_O), liquid ammonia solution (25% NH_3_), monosodium dihydrogen phosphate (NaH_2_PO_4_), hydrochloric acid (HCl), disodium hydrogen phosphate (Na_2_HPO_4_), N-methyl-2-pyrrolidone (NMP), potassium hydroxide (KOH), polyvinylidene fluoride (PVDF), and nifedipine (NFD) were bought from Sigma-Aldrich, USA. Nickel foam (NF) and carbon black (CB) were purchased from the Daejung Chemicals Company, Republic of Korea. For adjusting the pH, HCl and NaOH were utilized, while NaH_2_PO_4_ and Na_2_HPO_4_ salts were employed for making a 0.1 M phosphate-buffered solution (PBS). All of the reagents and deionized water were of analytical-grade quality and did not undergo any further preparation before being used.

### 3.2. Synthesis of CuSn(OH)_6_ Microspheres

CuSn(OH)_6_ microspheres were prepared using the coprecipitation method with slight modification from previously reported procedures [20]. First, a 0.06 M CuSO_4_·5H_2_O solution was prepared in 25 mL of deionized water and stirred continuously to ensure homogeneity. Then, 25% NH_3_ solution was added dropwise to the above solution until the blue color of the precipitate turned dark blue. This was followed by the dropwise addition of 0.06 M Na_2_SnO_3_.3H_2_O solution made in 25 mL of deionized water, which was stirred constantly for about 30 min. The collected precipitate was washed thoroughly with water and ethanol and dried at 60 °C for 12 h to obtain a powder form of pure CuSn(OH)_6_ phase.

### 3.3. Instrumentation

Various analytical techniques were employed to determine the physicochemical features of the as-developed material. The crystalline structure and phase purity of the perovskite sample were examined through X-ray diffraction (XRD) analysis, carried out on a Panalytical X’PERT PRO diffractometer (The Netherlands) with Cu Kα radiation (λ = 1.5406 Å) operating at 40 kV and 30 mA. The surface area and pore size distribution of CuSn(OH)_6_ were probed using nitrogen adsorption–desorption isotherm measurement on Micromeritics ASAP 2020 (USA). The experiment was conducted using field emission scanning electron microscopy (FESEM, TESCAN MIRA LMH, Czech Republic) and transmission electron microscopy (TEM, JEOL JEM-1010, Japan) coupled with energy-dispersive X-ray spectroscopy (EDS), which presented detailed information about the surface characteristics and elemental analysis of the as-proposed material. All electrochemical measurements, including cyclic voltammetry (CV), differential pulse voltammetry (DPV), electrochemical impedance spectroscopy (EIS), and galvanostatic charge–discharge (GCD), were performed through a CHI 660D electrochemical workstation (USA). A conventional three-electrode setup was utilized for the NFD sensor, consisting of a modified glassy carbon electrode (GCE) as the working electrode, a platinum wire as the counter electrode, and a saturated Ag/AgCl as the reference electrode. Similarly, in SCs, NF is the working electrode, a platinum wire is the counter electrode, and a Hg/HgO is the reference electrode.

### 3.4. Fabrication of CuSn(OH)_6_/GCE and CuSn(OH)_6_/NF

The GCE surface was meticulously polished using an alumina slurry to achieve a clean and contamination-free interface. After that, an even dispersion of CuSn(OH)_6_ (5 mg) in distilled water (1 mL) was created by vigorously mixing. To ensure strong adherence to the electrode substrate, an aliquot of CuSn(OH)_6_ suspension (6 μL) was gently drop-cast onto the pretreated GCE surface, followed by infrared (IR) drying for around 10 min. Subsequently, the CuSn(OH)_6_-modified GCE was successfully used as the working electrode for NFD sensing. Likewise, the NF substrate was washed with HCl for 15 min, then with deionized water for three washes. After washing, NF was dried in an oven at 80 °C and weighed. A slurry was made using PVDF, CB, and electroactive material in a 10:20:70 mass ratio. NF was covered with 3.5 mg of slurry using a micropipette and then dried at 80 °C for 12 h. This procedure ensured the preparation of CuSn(OH)_6_/NF electrode for SCs.

## 4. Conclusions

In summary, an effective and novel electrode material based on perovskite CuSn(OH)_6_ microspheres was synthesized via a coprecipitation method for dual electrochemical sensing and supercapacitor applications. Structural and morphological investigations validated the successful integration of the sphere architecture, which showed a homogenous distribution, increased surface texture, and distinctive structural characteristics beneficial to electrochemical reactions. Electrochemical analyses confirmed the improvement, with the perovskite electrode revealing noteworthy electrocatalytic activity toward NFD oxidation through a two-electron and two-proton process as compared to the bare electrode. Under optimized DPV parameters, the CuSn(OH)_6_/GCE exhibited superior analytical performance toward NFD, with a wide linear range (0.4–303.3 µM) and a low LOD (0.44 µM). This voltammetric sensor also showed good cycling stability (84.79% retention) and reproducibility (3.3% RSD), and demonstrated excellent accuracy in the serum sample with satisfactory recoveries (99.2–99.8%). Additionally, in the three-electrode configuration, electrochemical tests revealed that the CuSn(OH)_6_/NF electrode presented remarkable supercapacitive properties, achieving a high C_sp_ (514 F g^−1^), along with satisfactory durability (83.05%) after 5000 remitting cycles and low resistance values (R_ct_ = 6.56 Ω and R_s_ = 1.04 Ω). These results highlight the potential of perovskite bimetal hydroxide for electrochemical sensing and energy storage.

## Figures and Tables

**Table 1:** Comparison between CuSn(OH)_6_/GCE and previously modified electrodes for NFD detection.

Modified Electrode	Method	Electrolyte/pH	Behavior	Linear Range (µM)	LOD (µM)	Matrix	Reference
AgNPs–MGCE ^a^	DPV	PBS ^e^/pH 9	Reduction	0.8–60	0.72	Tablet & Urine	[9]
BDDE ^b^	DPV	TRIS ^f^/pH 8	Oxidation	3.98–107	0.612	Tablet	[10]
MWCNTs- COOH/PANI/ITO ^c^	CV	BR ^g^/pH 2	Oxidation	1–100	1.0	–	[11]
m-AgSAE ^d^	DPV	BR/pH 8	Reduction	2–20	1.2	Water	[12]
CuSn(OH)_6_/GCE	DPV	PBS/pH 7.4	Oxidation	0.4–303.3	0.44	Serum	This work

^a^ Ag-nanoparticle-modified glassy carbon electrode; ^b^ Boron-doped diamond electrode; ^c^ Carboxylic acid-functionalized multiwalled carbon nanotubes modified with polyaniline on ITO plates; ^d^ Mercury meniscus silver solid amalgam electrode; ^e^ Phosphate-buffered solution; ^f^ Tris(hydroxymethyl)aminomethane buffer solution; ^g^ Britton–Robinson buffer solution.

**Table 2:** Comparison between CuSn(OH)_6_/NF and previously modified electrodes for SCs.

Modified Electrode	Morphology	Electrode System	Electrolyte	Capacitance (F g^−1^)	Current Density (A g^−1^)	Reference
CuSn(OH)_6_	Particles	Three	1 M H_2_SO_4_	135	2 mV/s	[21]
CoSn(OH)_6_	Particles	Three	3 M KOH	364	0.5	[17]
CoSn(OH)_6_	Particles	Three	3 M NaOH	450	2 mV/s	[29]
MnSn(OH)_6_	Particles	Three	1 M KCl	512	0.4	[30]
MnSn(OH)_6_	Particles	Three	1 M Na_2_SO_4_	31.2	5 mV/s	[31]
Mn_2_SnO_4_	Cubic-like	Three	1 M Na_2_SO_4_	298	1 mA/cm^2^	[32]
CuSn(OH)_6_/NF	Spheres	Three	3 M KOH	514	1	This work

## Data Availability

The original contributions presented in this study are included in the article. Further inquiries can be directed to the corresponding authors.
